# Work History and Functional Limitations in Later Life Under Changed Institution in China

**DOI:** 10.1093/geroni/igaf122.190

**Published:** 2025-12-31

**Authors:** Chengming Han

**Affiliations:** UTMB Health, The University of Texas Medical Branch, Galveston, Texas, United States

## Abstract

**Objective:**

This study examines the impact of work history on functional limitations in later life among individuals who experienced China’s 1978 economic reform.

**Methods:**

Data were drawn from the 2011–2018 China Health and Retirement Longitudinal Study (CHARLS) for respondents born between 1934 and 1964. Sequence analysis was used to categorize work histories by type and duration. Tobit models were applied to estimate the effects of work trajectories on functional limitations in later life.

**Results:**

The economic reform facilitated transitions from farming to non-rural work, yet hukou-based urban-rural divisions shaped access to pensions, medical insurance, and housing benefits. Urban work histories were associated with lower functional limitations and a slower decline over time. Compared to urban workers with an urban hukou, self-employed individuals with an urban hukou (b = 1.570, p < 0.001), farmers with a rural hukou (b = 1.465, p < 0.05), and older migrant workers (b = 1.662, p < 0.001) exhibited higher levels of functional limitations.

**Conclusion:**

Rural hukou holders had fewer opportunities for non-rural employment, limiting their access to pensions, healthcare, and better living conditions. Those engaged in rural work experienced significantly higher functional limitations compared to individuals in other employment types.

